# Editorial: Hot topic: Reducing operating times and complication rates through robot-assisted surgery

**DOI:** 10.3389/frobt.2022.1046321

**Published:** 2022-10-10

**Authors:** Daniele Cafolla, Francesco Calimeri, Huiping Cao, Matteo Russo, Dominique Sappey-Marinier, Paolo Zaffino

**Affiliations:** ^1^ IRCCS Neuromed, Pozzilli, Italy; ^2^ University of Calabria, Cosenza, Italy; ^3^ New Mexico State University, Las Cruces, NM, United States; ^4^ University of Nottingham, Nottingham, United Kingdom; ^5^ Université Claude Bernard-Lyon1, University of Lyon, Lyon, France; ^6^ Magna Græcia University of Catanzaro, Catanzaro, Italy

**Keywords:** surgical robots, minimally-invasive surgery, robot-assisted surgery, complication rates, operating time

## 1 Introduction

Robotics is playing more and more an important role in medicine and surgery. The importance of the integration of robotic technologies in the medical field has only been amplified by the COVID-19 pandemic and the subsequent requirement for socially-distanced care and teleoperated medical assistance. Many recent developments in related fields, including, but not limited to, Artificial Intelligence (AI), machine learning, soft and continuum robotics, and teleoperation, are enabling robot-assisted surgery to maximize the efficiency and effectiveness of surgical operations while reducing invasiveness and potential complications.

A crucial question when considering robot-assisted medical intervention is whether the robot system is as effective, or even more so, than a human surgeon. Key indicators for this effectiveness include, but are not limited to, operating time and complication rate. This research topic aimed to build on the existing developments in robot-assisted surgery by exploring the role of robot systems in reducing operating times and complication rates, investigating the occurrences, causes and outcomes of surgical complications, and discussing how the robotics industry can address these issues for the future.

### 2 Contributions

This issue includes five contributions that address robot-assisted surgery from different perspectives, aimed at enhancing surgical performance with novel solutions for automatic endoscope guidance (Gruijthuijsen et al.), pre-operative planning (Lambrechts et al.), classification of clinical profiles (Barile et al.), robot base positioning (Sundaram et al.), and minimally-invasive ultrasound scanning (Marahrens et al.).

Gruijthuijsen et al. focus on bi-manual surgical operations, which usually require a second surgeon to maneuver an endoscope and provide visual feedback to the operating surgeon. While robotic endoscope holders have been proposed, existing prototypes impose an additional cognitive load on the now solo surgeon, hindering their clinical acceptance. Conversely, Gruijthuijsen et al. proposes a novel approach that combines tooltip localization with surgical tool segmentation and visual servoing providing synergistic interaction between surgeons and robotic endoscope holders. The system is validated with a bi-manual surgery case study.

Lambrechts et al. propose an AI-driven tool to improve surgeon and patient specific default preoperative plans for knee arthroplasty. As generic preoperative plans require frequent time-consuming changes, a predictive method is shown to reduce by almost 40% the average number of corrections required to adapt a generic plan to a specific patient. This study included over 5,400 operative plans, corrected by 39 surgeons.

Barile et al. use machine learning techniques to discriminate multiple sclerosis clinical profiles through grey matter thickness connectome data. Starting from a dataset of 90 multiple sclerosis patients with four distinct clinical profiles, the proposed pipeline achieves a successful classification in over 70% of the cases using six global graph metrics extracted from the grey matter morphological connectome of the patients. These promising results show the potential and efficiency of the proposed method when compared to complex MRI techniques.

Sundaram et al. aim at improving operation efficiency by optimizing the base location of surgical robots. The proposed method, based on robot capability maps, identifies the optimal positioning of a surgical robot by considering not only robot kinematics but also environmental constraints such as available access ports (e.g., for laparoscopy). This algorithm reduces setup time while improving the setup itself, thus increasing the acceptance of robot-assisted surgery by surgeons and clinical personnel.

Marahrens et al. address robotic ultrasound scanning invasiveness. Autonomous ultrasound scanning has been researched for over 2 decades, but minimally invasive operations are intrinsically limited by inaccurate force sensing and unreliable kinematics. In this work, these challenges are addressed with an attitude sensor fusion scheme for improved kinematic sensing and a visual deep-earning algorithm to ensure contact between the ultrasonic probe and the target surface.

### 3 Perspectives

The present collection confirms that the scientific community is actively working on robotics and AI for surgical procedures, thus producing a significant increase in performance and effectiveness of these technologies; in the broader context of healthcare, Robotics and Artificial Intelligence propose a wide range of tools and methods, and further achievements will be granted in the next future by studying proper combinations of such different results, integrating both hardware and software solutions.

